# COVID-19 Infection in Pregnancy: Obstetrical Risk Factors and Neonatal Outcomes—A Monocentric, Single-Cohort Study

**DOI:** 10.3390/vaccines10020166

**Published:** 2022-01-21

**Authors:** Antonella Vimercati, Rosalba De Nola, Paolo Trerotoli, Maria Elvira Metta, Gerardo Cazzato, Leonardo Resta, Antonio Malvasi, Archiropita Lepera, Ilaria Ricci, Manuela Capozza, Nicola Laforgia, Ettore Cicinelli

**Affiliations:** 1 Unit of Obstetrics and Gynecology, Department of Biomedical and Human Oncologic Science, University of Bari, 70124 Bari, Italy; antonella.vimercati@uniba.it (A.V.); antoniomalvasi@gmail.com (A.M.); leperaa@libero.it (A.L.); ilaria.ricci92@libero.it (I.R.); ettore.cicinelli@uniba.it (E.C.); 2 Medical Statistics, Department of Biomedical and Human Oncologic Science, University of Bari, 70121 Bari, Italy; paolo.trerotoli@uniba.it (P.T.); maria.metta@uniba.it (M.E.M.); 3 Department of Emergency and Organ Transplant, University of Bari, 70121 Bari, Italy; gerycazzato@hotmail.it (G.C.); leonardo.resta@uniba.it (L.R.); 4 Unit of Neonatology and Intensive Care, Department of Biomedical and Human Oncologic Science, University of Bari, 70100 Bari, Italy; manuela.capozza@uniba.it (M.C.); nicola.laforgia@uniba.it (N.L.)

**Keywords:** COVID-19, SARS-CoV-2, pregnancy, neonate, neonatal respiratory distress syndrome (RDS), preterm birth (PTB), delivery

## Abstract

The effects of coronavirus disease 2019 caused by severe acute respiratory syndrome coronavirus 2 on pregnant women and neonates are mainly unknown, since limited data are available in the literature. We conducted a monocentric and cross-sectional study enrolling 122 un-vaccinated pregnant women with COVID-19 infection tested by RT-PCR nasopharyngeal swab. Only 4.1% of the patients had severe COVID-19 symptoms together with major respiratory symptoms and intensive care unit admission, whereas 35.25% of women had comorbidities and two-thirds of them were overweight or obese. COVID-19 was detected mainly in the third trimester (98.36%) and multiparous women (59.02%). The mode of delivery was influenced by mild–severe COVID-19 symptoms, with a higher number of urgent or emergent cesarean sections than spontaneous or operative vaginal births. Preterm births were associated with high BMI, mode of delivery (higher among cesarean sections), nulliparity, and severe COVID-19 symptoms. In cases of severe COVID-19 symptoms, there was a higher rate of respiratory distress syndrome among newborns. In the end, only the presence of a severe COVID-19 infection worsened the obstetrical and neonatal outcomes, with higher rates of urgent or emergent cesarean section, preterm births, and neonatal respiratory distress syndrome.

## 1. Introduction

The coronavirus disease 2019 (COVID-19) pandemic caused by severe acute respiratory syndrome coronavirus 2 (SARS-CoV-2) began in December 2019 in Wuhan, Hubei Province, China, and then rapidly spread worldwide until declared a Public Health Emergency of International Concern (PHEIC) by the Pan American Health Organization and World Health Organization (PAHO/WHO) on the 30th of January [1].

Even if a huge amount of information is available regarding the epidemiology, biology, and clinical presentation of COVID-19 infection, limited data are available in the global literature regarding the immune response during pregnancy, the effects of possible vertical transmission through a placental infection, inflammation, and the fetoplacental defense tools in cases of maternal COVID-19 infection [2].

Little is known about COVID-19 in pregnancy, although the epidemiological data reported by the Centers for Disease Control and Prevention (CDC) are alarming; in the case of COVID-19 infection, 31.5% of pregnant women underwent hospitalization vs. 5.8% of non-pregnant women in June 2020 [3]. In detail, the maternal and perinatal outcomes after a COVID-19 infection during pregnancy have been mainly limited to systematic reviews, case reports, and case series [2,4,5,6,7].

This monocentric and cross-sectional study enrolled 122 pregnant women admitted to the regional COVID-19 “hub” hospital (Obstetrics Division) for health issues related to COVID-19 or the asymptomatic findings of nasopharyngeal RT-PCR swab, as routinely performed near the due date of delivery.

## 2. Materials and Methods

This was an observational, prospective, monocentric, single-cohort study that principally aimed to evaluate the maternal and perinatal outcomes of COVID-19 infection during pregnancy, involving 193 women that came to our attention for their COVID-19 results based on nasopharyngeal RT-PCR swab, as routinely performed at the time of admittance at the Gynaecological and Obstetrical Division of a regional COVID-19 “hub” hospital, and before each assessment in the emergency room from August 2020 to June 2021, covering the second and third pandemic waves that peaked on November 2020 and March 2021, respectively.

The present study was conducted according to principles and standards of the Good Clinical Research Practice after Ethical Committee approval (IRB approval n. 190/CS, 28 January 2021) and the acquisition of informed consent from all the patients.

Inclusion criteria: pregnant women with COVID-19 infection evaluated as positivity for COVID-19 the time of nasopharyngeal RT-PCR swab, aged 18 years old and over, informed consent, pregnant patients who delivered at our center.

Exclusion criteria: non-pregnant patients, pregnant women without COVID-19 infection evaluated as negativity for COVID-19 the time of nasopharyngeal RT-PCR swab, women aged less than 18 years old, without informed consent, pregnant patients who delivered at a different center, miscarriage, and incomplete data for 4 or more entries.

After the hospital discharge, the medical records were sanitized for data collection purposes. The general characteristics of the population under study are summarized in Table 1.

Data about symptoms were missing in only 0.82% of the population. Regarding the mothers’ characteristics, we collected the following data: COVID-19 symptoms (no, low, mild, severe), age (then classified in two classes: <35/≥35 years), Body Mass Index (BMI), ethnicity, parity, gestational age (G.A.) at the time of COVID-19 infection, comorbidities, preterm premature rupture of membranes (pPROM), premature rupture of membranes (PROM), mode of delivery-spontaneous vaginal birth (SVB), operative vaginal birth (OVB), elective/urgent/emergent cesarean section (CS).

In detail, the comorbidities included obesity (BMI > 30 kg/m^2^), diabetes (diabetes mellitus, gestational diabetes), hypertension and pre-eclampsia, and other miscellaneous ones. Notably, maternal age >35 years old was considered a risk factor. COVID-19 symptoms were classified into four categories, as follows: asymptomatic (group 1), few symptoms (group 2A), mild symptoms (group 2B, up until major symptoms and dyspnoea requiring non-invasive respiratory support with 02-mask), and severe symptoms with pneumonia requiring ICU admission (group 3). Groups 2A and 2B mainly experienced fever, shivering, weakness, myalgia, arthralgia, dry cough, anosmia, ageusia, nasal congestion, sore throat, and dyspnoea. Rarely, patients reported diarrhea, sickness, vomiting, and dyspnoea.

Regarding neonates, we considered preterm birth (PTB) and full-term birth to be before and above 37 weeks, respectively, whereas the birth weight was classified as small for GA (SGA), appropriate for GA (AGA), or large for GA (LGA) if neonatal weight was <10° percentile, 10° ≤ percentile < 95°, or >95° percentile, respectively, according to the growth chart from the World Health Organization [8]. The PTB group was divided into three subgroups, as shown in Table 1. Moreover, we described eventual malformations, respiratory distress syndromes (RDSs), neonatal intensive care unit access (NICU), and eventual positivity to COVID-19.

Firstly, we characterized the population of pregnant women with COVID-19 infection and the relative neonates, then we analyzed the possible correlations of severe maternal COVID-19 infection with obstetrical population characteristics, defining possible risk factors for obstetrical and neonatal outcomes (i.e., PTB and RDS) as the main goals of the study.

All data were collected and analyzed as categorical variables. All variables were summarized as counts and percentages and comparisons between independent groups were performed by chi-square or Fisher’s exact test, as appropriate. The difference in percentage and the 95% confidence interval were reported together with the *p*-value. An adjustment of *p*-values was used for multiple comparisons through a permutational adjustment for Fisher’s exact test by applying Proc Multtest by SAS 9.4 for PC. Here, *p*-values < 0.05 were considered for statistical significance. The software used to manage and analyze data was SAS 9.4 for PC.

To determine the sample size, the risk of preterm birth (see point 2 of the previously described goals) and the risk of neonatal RDS (see point 3) were considered.

The percentage of preterm birth is 6.7% in Italy [9], and it was supposed that in women with severe COVID-19, the percentage could reach 15%. To estimate an odds ratio equal to 2, with stated type I error and type II error equal to 0.05 and 0.2, respectively, the total sample size should be 105.

The prevalence of RDS is estimated in 1% of all newborns [10], and it was supposed that the percentage of RDS in children born from mothers affected by severe COVID-19 could be at least 10%. To estimate an odds ratio equal to 2 with type I error and type II error equal to 0.05 and 0.2, respectively, the total sample size should be 139 newborns. The sample size was determined by the Demidenko method using the software G*Power version 3.1.9.2.

## 3. Results

### 3.1. Results from Descriptive Statistics of the Population under Study (Mothers and Neonates)

1. General characteristics of the population under study and main neonatal outcomes

We enrolled 122 out of 193 patients, since they met all the inclusion criteria. In detail, we excluded 71 patients as follows: 22 deliveries (different centers or incomplete data), 21 miscarriages, 2 ectopic pregnancies, 9 cases of COVID-19 infection during the puerperium, and 17 non-pregnant patients who came to our attention for gynecological issues. The main characteristics data are summarized in Table 1 and Table 2.

2. COVID-19 symptoms among the population under study

Most of the patients had an asymptomatic infection (group 1, 60%), whereas the remnants (40%) had a spectrum of symptoms from mild to severe with hospitalization (groups 2A, 2B, and 3) (see Table 1). Furthermore, most of the clinical records (95.0%) covered groups 1, 2A, and 2B, whereas the presence of severe symptoms with severe respiratory symptoms requiring ICU admission was registered only in 4.1% of cases (see Table 1). However, we registered only two cases of patients intubated without any case of maternal death.

3. Comorbidities of the population under study

Here, 35.25% of the population under study had comorbidities, such as obesity (BMI > 30 kg/m^2^), diabetes (diabetes mellitus, gestational diabetes), hypertension or pre-eclampsia, and other miscellaneous comorbidities; maternal age > 35 years old was considered a risk factor. Notably, more than two-thirds of women were overweight or obese (56.56% and 20.49%, respectively). The diagnosis of COVID-19 emerged mainly in the third trimester (98.36%), with a higher rate in the multiparous woman (59.02%).

4. Rates of different modes of delivery, preterm birth, and neonatal respiratory distress syndrome

The overall (elective, urgent, emergent) CS rate was 38.52%, whereas the SVB accounted for 55.74% and OVB for 2.46% of the deliveries (missing data only for 3.28% women). None of the pregnant women were vaccinated. Once the population of pregnant women with COVID-19 infection was defined and described, we evaluated the findings for the relative neonates (Table 1). Overall, the rate of PTB was 10.4%, with a majority of late PTB (8%). The rate of RDS was 5.6%. The NICU admittance was 10.4%, even if none of the neonates were positive for COVID-19 infection (Table 2).

### 3.2. Results from Correlations between Maternal or Obstetric Risk Factors and COVID-19 Disease Severity, Gestational Age at Birth, and Neonatal Respiratory Distress Syndrome

1. The correlation between maternal/obstetric risk factors and COVID-19 disease severity

Therefore, we analyzed the possible correlation between COVID-19 symptoms and obstetrical population characteristics (maternal age, BMI, ethnicity, comorbidities, G.A. at COVID-19 diagnosis, parity, delivery mode), defining possible risk factors for adverse obstetrical outcomes (Table 3).

Notably, the mothers’ COVID-19 symptoms (groups 1 + 2A vs. groups 2B + 3) correlated significantly (OR = 8.4, 95% CI 2.7–25.6%, *p* = 0.0001) with the mode of delivery (Table 3). After the post-hoc analysis with the *p*-value adjustment, the comparisons for each mode of delivery (SVB, OVB, elective/urgent/emergent CS) revealed a statistically significant difference about urgent and emergent CS (33.5%, 95% CI 14.5–54%, *p* = 0.002) between the subset 2A + 2B (7.37%) and group 3 (40.91%). The COVID-19 symptoms worsened for women aged 35 or over (23.08%, 95% CI 2.24–43.71%, *p* = 0.02) (see Table 3). With the aim of evaluating the effects of maternal characteristics (maternal age, BMI, ethnicity, comorbidities, G.A. at COVID-19 diagnosis, parity, delivery mode) and COVID-19 symptoms on neonatal outcomes (birth age, birth weight, respiratory distress syndrome (RDS), malformations, neonatal intensive care), we found significant results only for PTB (Table 4) and RDS (Table 5). In other words, the presence of severe COVID-19 infection increased the rate of urgent or emergent cesarean sections, preterm births, and neonatal respiratory distress syndrome.

2. Maternal characteristics and neonatal birth age (full-term vs. preterm birth)

**Figure 1:**
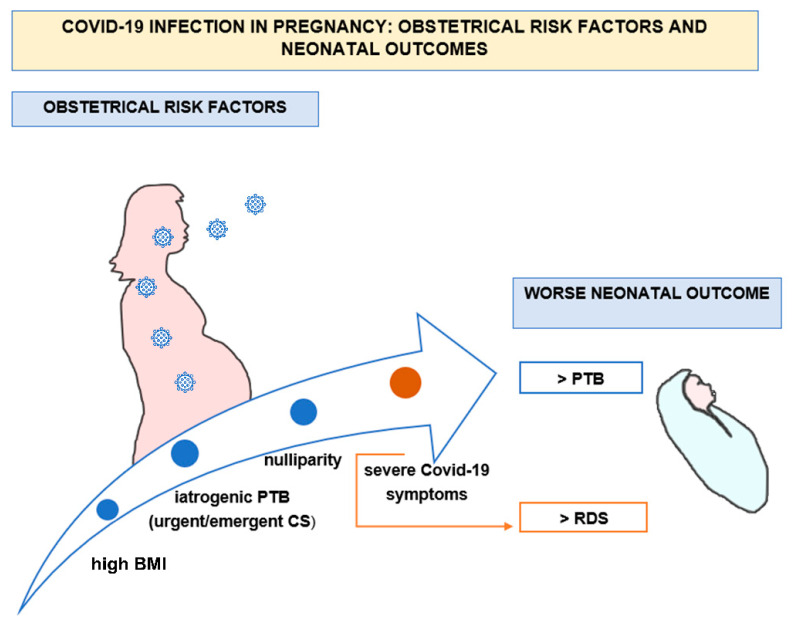
COVID-19 infection during pregnancy influences the neonatal outcome, increasing the rate of preterm birth (PTB) due to high BMI, iatrogenic causes of PTB requiring urgent or emergent cesarean section (CS), and the clinical pattern of severe maternal COVID-19 symptoms. In the last condition, there is also a higher risk of neonatal respiratory distress syndrome (RDS).

Overall, preterm births (PTBs) covered 10.4% of deliveries (see Table 2). Interestingly, there was a statistically significant association between BMI and PTB vs. term birth (*p* = 0.01); after adjusting for multiple comparisons, the percentage of obese patients was higher in full- and preterm births (difference 16.65%, 95% CI −2.86 to 46.45%, *p* = 0.03) (see Table 4). As expected, the relationship between the delivery mode and PTB (Table 4) was also statistically significant (*p* = 0.0005), and in multiple comparisons the percentage SVBs was significantly different between PTBs and full-term births (difference 24.1%, 95% CI −3.8 to 45.37%), such as in emergent CS (19.41%, 95% CI −7.29 to 30.5) or urgent CS (43.57, 95% CI 17.84 to 66.94). The percentages of other comorbidities were significantly higher in PTB than in full-term groups (difference 40.98, 95% CI 16.57 to 62.75, *p* = 0.003). Notably, PTBs were more frequent for nulliparous women (difference 36.33%, 95% CI 7.45 to 53.77, *p* = 0.01) and with the presence of severe COVID-19 symptoms (30.12%, 95% CI 5.76 to 55.48, *p* = 0.01) (see Table 4 and Figure 1). Gestational age at birth, however, was not influenced by ethnicity (not shown) or gestational age at maternal COVID-19 infection (Table 4).

3. The correlation between the presence of neonatal respiratory distress syndrome (RDS) and the severity of maternal COVID-19 symptoms

The overall rate of respiratory distress syndrome (RDS) for newborns was 5.6% (Table 2). In detail, the RDS frequency significantly increased only when the mother experienced severe COVID-19 symptoms (difference 38.79%, 95% CI 5.64 to 66.52, *p* = 0.03) (see Table 5 and Figure 1).

4. Bad neonatal outcomes

We recorded three cases of fetal malformations judged as independent from maternal COVID-19 infection (Table 2). We did not register any stillbirth or neonatal death. The size of the newborns appeared to be significantly correlated only with ethnicity (*p* = 0.006), without a clear or statistically significant correlation of a specific ethnic group with the highest frequency of SGA or LGA.

## 4. Discussion

Limited data are available regarding the fetal and maternal effects of COVID-19 infection. The results of this study depicted a risk profile associated with obstetrical and neonatal adverse outcomes associated with COVID-19 during pregnancy.

The population enrolled was composed of COVID-19-infected pregnant women, who were mainly asymptomatic and oligosymptomatic. In detail, we found that 80% of cases were asymptomatic or showed few symptoms, 15% showed mild–severe symptoms, and only 4% of admissions to ICUs were for severe symptoms, with similar rates as in the literature [2,7]. In our cohort, the diagnosis was made mostly in the third trimester (98.36%), with only two cases during the second trimester (1.64%), with rates in the literature ranging approximately 86–90% and 14–7%, respectively [2,7,11].

In the population under study, we found a high positive correlation between age >35 years and COVID-19 symptoms, with a non-significant positive trend between BMI and COVID-19 symptoms. It is known that the COVID-19 infection worsens in case of advanced maternal age, obesity, Hispanic or Latino origin, and other medical comorbidities [11]. From a biological point of view, the presence of comorbidities (diabetes, preeclampsia, cardiovascular disease, and hypertension) or older age is associated with reduced production of the protective peptide angiotensin-(1–7) by the membrane-bound angiotensin-converting enzyme 2 (ACE2), leading to vasoconstriction, inflammation, fibrosis, edema, and lung damage [12].

In our cohort, the overall rate of neonatal RDS was 5.6%. Strikingly, the frequency of RDS increased significantly only in cases of severe COVID-19 infection (*p* = 0.03). Notably, 10.4% of newborns were admitted to the NICU, with a higher rate of admission in cases of severe COVID-19 symptoms. In the literature, the rate of neonates admitted to NICU is higher in mothers with COVID-19 pneumonia (27.1%) than controls (10.1%) [7]. We did not register any stillbirth or neonatal death, in line with the epidemiologic evidence that the rates of stillbirths (0.7%) and neonatal deaths (0.2%) were similar between the first semester of 2020 and the previous four years [3,7,9,13,14,15,16].

We found a highly statistically significant association between the rate of urgent or emergent CS and the onset of severe COVID-19 infection. The severity of COVID-19-related symptoms of the mother, especially respiratory distress and subsequent fetal hypoxia, determines the eventual invasive procedures (iatrogenic CS) and the delivery-timing decision, which must consider the possible negative neonatal outcomes due to prematurity [6] and the mode of delivery.

Interestingly, we found a significantly higher risk of PTB in the presence of severe COVID-19 symptoms (*p* = 0.01), miscellaneous comorbidities (*p* = 0.003), nulliparity (*p* = 0.01), and BMI > 25 (*p* = 0.034), with a collective rate of PTB of 10.6%, lower than the findings of a recent metanalysis (PTB rate of 29.7–16%) [17] but higher than the PTB rate before the pandemic in Italy (6.7%) [9]. However, PTB and severe COVID-19 share many risk factors, such as body mass index > 24.9, asthma, and chronic hypertension [18]. A recent study found that the proportion of PTB (mostly late PTB) increased during the wild-type and subsequent alpha waves of COVID-19 in Italy to 10.9% (vs. 6.7% in 2019), rocketing to 42.9% during the alpha wave among women affected by pneumonia (*p* < 0.001).

The risk of PTB (<37, <34, and <28 weeks) is related to the severe maternal COVID-19 infection with placental villous oedema [3]. Frequent histological patterns of COVID-19-infected placenta were found during a large double-blinded case–control study (71:142) [18], as follows: fetal vascular malperfusion (FVM) (21.1% vs. 4.2% *p* < 0.001), arteriopathy (40.9% vs. 1.4% *p* < 0.0001), decidual inflammation (32.4% vs. 0.7% *p* < 0.0001), perivillous fibrin deposition (36.6% vs. 3.5% *p* < 0.0001), fetal vessel thrombi (22.5% vs. 0.7% *p* < 0.0001), adaptational maternal vascular malperfusion (MVM) (54.3% vs. 43.7% *p* = 0.19) [19].

The localization of ACE2 (SARS-CoV-2 receptor) is weak on the stromal side of the villous syncytiotrophoblast, as is the expression of transmembrane serine protease 2 (TMPRSS2, activating cofactor for viral cell entry) on the maternal endothelium [2], partially explaining the extremely rare vertical transmission [1,15,20,21,22,23]. The maternal infection can cause viremia leading to placental infection and microangiopathy leading to vertical transmission in exceptional cases.

In our cohort, all newborns were negative for COVID-19 infection at the time of nasopharyngeal PCR swab. A recent multicentric Italian study showed that the nasopharyngeal PCR positivity was extremely rare (0.0–6.0%) [5].

The absence of vaccinated and control pregnant patients and the consideration of only pandemic waves were the main limits of our study; thus, it might be useful to evaluate the correlations of maternal–fetal outcomes with new viral variants and the role of the vaccine during pregnancy.

## 5. Conclusions

Pregnancy can be complicated in cases of severe COVID-19 infection (4%) and needs to be evaluated at specialized centers that can manage complex cases using multidisciplinary teams. Here, 35.25% of the population under study had comorbidities (hypertension, pre-eclampsia, diabetes mellitus, gestational diabetes, and other miscellaneous comorbidities), with two-thirds being overweight or obese. The diagnosis of COVID-19 was made mainly in the third trimester and in multiparous women. The mode of delivery was influenced by the presence of mild–severe COVID-19 symptoms, with a higher number of urgent or emergent CS than SVB or OVB. Birth age (full-term vs. PTB) was not influenced by ethnicity or gestational age at maternal COVID-19 infection. Overall, preterm births were associated with high BMI, mode of delivery (higher among cesarean sections), nulliparous women, and severe COVID-19 symptoms. Only in cases of severe COVID-19 symptoms did we register a higher rate of respiratory distress syndrome among newborns.

## Figures and Tables

**Table 1:** General characteristics of the population under study, including age, BMI, ethnicity, comorbidities, parity, gestational age (G.A.) at COVID-19 diagnosis, COVID-19 symptoms, pregnancy outcome, and mode of delivery. In the case of complete data for a variable, the relative row of “missing data” is not reported. The abbreviations used in the table are the same as in the manuscript.

General Characteristics of COVID-19 + Pregnant Patients		N	%
		122	100%
Age	<30	38	31.15%
	30–40	76	62.30%
	>40	7	5.74%
	Missing	1	0.82%
BMI	<25 normal and underweight	20	16.39%
	25–30 overweight	69	56.56%
	>30 obese	25	20.49%
	Missing	8	6.56%
Ethnicity	Caucasian	116	95.08%
	African	5	4.10%
	Asian	1	0.82%
Comorbidities	Hypertension/pre-eclampsia	2	1.64%
	Diabetes mellitus/gestational diabetes	12	9.84%
	Others	29	23.77%
	Healthy	79	64.75%
	Missing	1	0.82%
Parity	Nulliparous	50	40.98%
	Multiparous	72	59.02%
G.A. at COVID-19 diagnosis	1st trimester	0	0.00%
	2nd trimester	2	1.64%
	3rd trimester	120	98.36%
COVID-19 symptoms	1 none	74	60.66%
	2A few	24	19.67%
	2B mild	18	14.75%
	3 severe, requiring ICU for COVID-19 treatment	5	4.10%
	Missing	1	0.82%
Pregnancy outcome and mode of delivery	PROM	7	5.74%
	pPROM	0	0.00%
	SVB	66	54.10%
	OVB	3	2.46%
	elective CS	29	23.77%
	urgent/emergent CS	17	13.93%

**Table 2:** Main outcomes of the neonates born from the population under study, including birth weight, malformations, RDS, NICU admission, and neonatal nasopharyngeal PCR swab for COVID-19 infection (COVID-19 + neonates). In case of complete data for the variable, the relative row of “missing data” is not reported. The abbreviations used in the table are the same as in the manuscript. The percentages considered the missing data, which were not reported for reasons of clarity.

Main Neonatal Outcomes		Total Newborns = 125	
Gestational age at birth	Full-term 37–42	107	85.60%
	Late PTB 32–37	10	8.00%
	Early PTB 28–32	2	1.60%
	Extreme PTB < 28	1	0.80%
	Missing	5	4.00%
Birth weight	AGA	97	77.60%
	SGA	8	6.40%
	LGA	9	7.20%
	Missing	11	8.80%
Malformations		3	2.40%
RDS		7	5.60%
NICU admission		13	10.40%
COVID-19 + Neonates		0	0.00%

**Table 3:** The correlations between maternal or obstetric risk factors and COVID-19 disease severity. LEGEND: OVB (operative vaginal birth), SVB (spontaneous vaginal birth), CS (cesarean section); *: subgroups significantly different *p* < 0.001; n.s.: not statistically significant; *p* > 0.05. The percentages considered the missing data, which were not reported for reasons of clarity.

Maternal/Obstetric Risk Factors		COVID-19 SYMPTOMS				*p*-Value
		NO or FEW		MILD or SEVERE		
		N	%	N	%	
AGE	<35 yrs	73	75.26	12	52.17	0.02
	≥35 yrs	24	24.74	11	47.83	
BMI	<25 normal	18	20.00	2	8.7	0.17
	25–30 overweight	55	61.11	13	56.52	
	>30 obese	17	18.89	8	34.78	
ETHNICITY	Caucasian	93	94.90	22	95.65	n.s.
	African	5	5.10	0	0.00	
	Asian	0	0.00	1	4.35	
COMORBIDITY	hypertension/pre-eclampsia	1	1.02	1	4.35	n.s.
	diabetes/gestational diabetes	8	8.16	4	17.39	
	others	22	22.45	7	30.43	
	healthy	67	68.4	11	47.83	
GA AT COVID-19 DIAGNOSIS	1st trimester	0	0.00	0	0.00	n.s.
	2nd trimester	2	2.04	0	0.00	
	3rd trimester	90	97.96	23	100.00	
PARITY	nulliparous	42	42.86	8	34.78	n.s.
	multiparous	56	57.14	15	65.22	
DELIVERY MODE	OVB	2	2.11	1	4.55	0.0001
	SVB	59	62.11	9	40.91	
	elective CS	27	28.42	3	13.64	
	emergent CS	7	7.37 *	9	40.91 *	

**Table 4:** Maternal characteristics and gestational age at birth (full-term vs. preterm birth). LEGEND: OVB (operative vaginal birth), SVB (spontaneous vaginal birth), CS (cesarean section); *, @, #, §, a, b, c: subgroups significantly different *p* < 0.001; n.s.: not statistically significant, *p* > 0.05. The percentages considered the missing data, which were not reported for reasons of clarity.

Maternal/Obstetric Risk Factors		GESTATIONAL AGE AT BIRTH				*p*-Value
		FULL-TERM		PRETERM		
		N	%	N	%	
AGE	<35 yrs	75	61.54	8	70.09	n.s.
	≥35 yrs	32	38.46	5	29.91	
BMI	<25 normal	16	15.84	5	34.46	0.03
	25–30 overweight	66	65.35 *	3	23.08 *	
	>30 obese	19	18.81 @	5	38.46 @	
COMORBIDITY	hypertension/pre-eclampsia	1	0.93	1	7.69	0.003
	diabetes/gestational diabetes	8	7.48	3	23.08	
	others	22	2.56 #	8	61.54 #	
	healthy	76	71.03	1	7.69	
PARITY	Nulliparous	41	38.32 §	10	76.92 §	0.01
	multiparous	66	61.68	3	23.08	
GA AT COVID-19 DIAGNOSIS	1st trimester	0	0.00	0	0.00	n.s.
	2nd trimester	0	0.00	1	7.69	
	3rd trimester	107	100.00	12	92.31	
DELIVERY MODE	OVB	3	2.80	0	0.00	0.0005
	SVB	64	59.81	5	38.46	
	Elective CS	29	27.1 a	1	7.69 a	
	Emergent CS	11	10.28 b	7	53.85 b	
COVID-19 SYMPTOMS	NON OR FEW	89	83.96	7	53.85	0.01
	MILD OR SEVERE	17	16.04 c	6	46.15 c	

**Table 5:** The correlations between the presence of neonatal respiratory distress syndrome (RDS) and the severity of maternal COVID-19 symptoms. LEGEND: OVB (operative vaginal birth), SVB (spontaneous vaginal birth), CS (cesarean section); *: subgroups significantly different *p* < 0.001; n.s.: not statistically significant, *p* > 0.05. The percentages considered the missing data, which were not reported for reasons of clarity.

Maternal COVID-19 Symptoms	NEONATAL RDS				*p*-Value
	PRESENCE		ABSENCE		
	N	%	N	%	
none or few	3	42.86%	89	81.65%	0.03
mild or severe	4	57.14% *	20	18.35% *	
